# Incidental Visualization of Gallbladder on Post-therapy [ ^177^ Lu]Lu-DOTATATE Scintigraphy Mimicking a Liver Metastasis in a Duodenal Neuroendocrine Tumor

**DOI:** 10.1055/s-0044-1788073

**Published:** 2024-07-04

**Authors:** Parth Baberwal, Rahul Parghane, Sandip Basu

**Affiliations:** 1 Radiation Medicine Centre, Bhabha Atomic Research Centre, Mumbai, Maharashtra, India; 2 Homi Bhabha National Institute, Mumbai, Maharashtra, India

**Keywords:** neuroendocrine tumor (NET), ^68^ Ga-DOTATATE; [ ^177^ Lu]Lu-DOTATATE, PET-CT, SPECT-CT, somatostatin receptor (SSTR), peptide receptor radionuclide therapy (PRRT)

## Abstract

We present a rare case of physiological uptake of [ ^177^ Lu]Lu-DOTATATE in the gallbladder, observed post-therapy, in a 47-year-old man with grade I duodenal neuroendocrine tumor (NET), despite no uptake being observed pre-therapy in the somatostatin receptor-positron emission tomography. On planar scintigraphy, the gallbladder uptake could have been misidentified as liver metastasis. By utilizing single photon emission computed tomography/computed tomography imaging, we were able to precisely localize the tracer and obtain anatomical morphological characteristics, thereby averting the potential for misinterpretation of liver metastasis resulting from the gallbladder's physiological uptake of [ ^177^ Lu]Lu-DOTATATE in NET patients.

## Introduction

The efficacy of peptide receptor radionuclide therapy (PRRT) in the treatment of neuroendocrine tumors (NETs) is well recognized. Following the favorable results of the NETTER-1 trial, [177Lu]Lu-DOTATATE has emerged as a promising therapeutic radiopharmaceutical in clinical practice. 1 An essential requirement for PRRT is sufficient somatostatin receptor (SSTR) expression in NET, which is evaluated via SSTR imaging with the help of [ ^68^ Ga] Ga-DOTA-NOC/TATE positron emission tomography (PET)/computed tomography (CT). We present a case of grade I duodenal NET that had a localized tracer uptake in the gallbladder on a posttherapy [ ^177^ Lu]Lu-DOTATATE scan following PRRT. However, there was no SSTR uptake in the gallbladder region of the liver on a pre-therapy [ ^68^ Ga]Ga-DOTATATE PET/CT scan, which led to misinterpretation regarding the presence of new-onset liver metastatic disease.

## Case Report

**Fig. 1:**
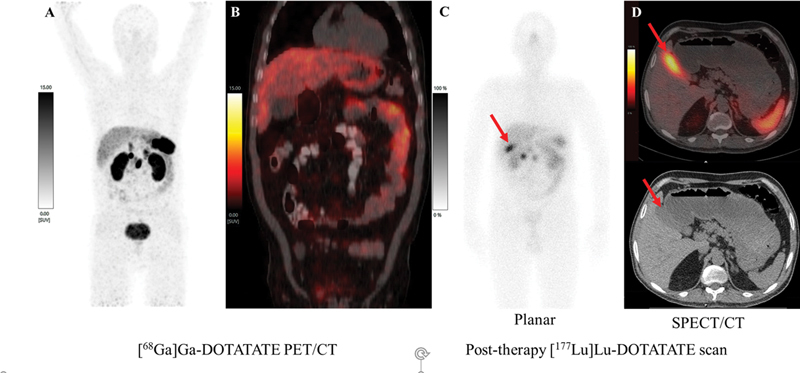
Maximum intensity projection ( **A** ) and coronal images ( **B** ) of fused pre-therapy [ ^68^ Ga]Ga-DOTATATE positron emission tomography (PET)/computed tomography (CT) showed somatostatin receptor avid duodenal lesions and no abnormal uptake in liver. post-therapy [ ^177^ Lu]Lu-DOTATATE with planar ( **C** ) image showed tracer activity in duodenal lesions and right inferior region of liver (red arrow) and single photon emission CT/CT (SPECT/CT) ( **D** ) of posttherapy [ ^177^ Lu]Lu-DOTATATE axial fused and axial CT images showed tracer activity localizing to the gallbladder (red arrows).

A 47-year-old gentleman with no comorbidities, presented with complaint of dyspepsia. Patient underwent abdominal ultrasound, which showed a hypoechoic lesion of size 3.5 × 3.0 cm near the body of pancreas. Ultrasound-guided biopsy of lesion showed grade 1 NET (mib-1 labeling index of 1–2%) expressing synaptophysin and chromogranin. Serum chromogranin A level was 4656 µg/L. The patient did not receive any long-acting octreotide. He was referred to our institute for [ ^68^ Ga]Ga-DOTATATE PET/CT imaging and further management. [ ^68^ Ga]Ga-DOTATATE PET/CT imaging was found to have additional SSTR expressing metastatic retroperitoneal and mesenteric lymph nodes and the duodenal lesion, was decided to treat with PRRT in view of metastatic disease. Pre-PRRT [ ^68^ Ga]Ga-DOTATATE PET/CT showed ( Fig. 1A and B ) SSTR expressing duodenal lesion (maximum standardized uptake value [SUVmax] 21.08) and SSTR expressing mesenteric and retroperitoneal lymph nodes (SUVmax 122.45) and no abnormal SSTR uptake in the liver. The patient received first cycle of PRRT with 6.58 GBq (178 mCi) activity of [ ^177^ Lu]Lu-DOTATATE. posttherapy [ ^177^ Lu]Lu-DOTATATE scan with planar image acquired at 24-hour postadministration of the radiopharmaceutical ( Fig. 1C ) showed tracer uptake in duodenal lesions, mesenteric, and retroperitoneal lymph nodes and abnormal focal tracer uptake in the right inferior region of liver which was new compared with pre-therapy [ ^68^ Ga]Ga-DOTATATE PET/CT scan and localized to the gallbladder on the single photon emission CT/CT (SPECT/CT) imaging without any morphological changes on CT image of SPECT/CT images ( Fig. 1D ). Further delayed acquisitions were not done due to logistical issues.

## Discussion

An important benefit of [ ^177^ Lu]Lu-DOTATATE PRRT is the ability to perform post-therapy imaging using a gamma camera, since ^177^ Lu emits gamma rays. This is an integral component used in theranostics, which involves assessing the SSTR uptake in NET using SSTR-PET/CT imaging, as well as visualizing the uptake of therapeutic radiopharmaceutical in tumors using post-treatment scintigraphy. The uptake of [ ^177^ Lu]Lu-DOTATATE by the tumor is essential for PRRT to be therapeutically effective in NET. Nevertheless, scintigraphy reveals that the pituitary gland, thyroid, pancreatic islet cells, adrenal glands, liver, spleen, kidneys, urinary bladder, and gut exhibiting physiological uptake of [ ^177^ Lu]Lu-DOTATATE. Thus, it is crucial to distinguish and differentiate this physiological uptake from the tumor uptake of [ ^177^ Lu]Lu-DOTATATE on scintigraphy, as the physiological uptake does not contribute to the therapeutic benefits of PRRT. Instead, it will cause diagnostic dilemmas and confusion for tumor response assessment and side effects in NET. 1 Incidental gallbladder visualization on scintigraphy is usually the result of physiological uptake because of the tracer excretion into the bile and rapid clearance of gallbladder activity may be seen after ingestion of a fatty meal. This has been documented in the literature, primarily for [ ^111^ In]In-pentetreotide scintigraphy. 2 3 Fortunately, there is no report of physiological visualization of the gallbladder using SSTR-PET/CT imaging due to the low SSTR uptake in the gallbladder. The occurrence of physiological uptake of [ ^177^ Lu]Lu-DOTATATE in the gallbladder is extremely uncommon. So far, only two reports have been reported in the literature. 4 5 Diekmann et al performed a delayed imaging 1 hour after fatty meal administration in a case of gallbladder visualization on post-[ ^177^ Lu]Lu-DOTATATE therapy scan. 4 Fatty meal cleared gallbladder activity and this method is useful in places where SPECT/CT facilities are not available. An additional possible explanation for abnormal uptake of [ ^177^ Lu]Lu-DOTATATE in the gallbladder is inflammation, given that SSTR expression has been observed in inflammatory pathologies. 6 7 8 In our case, the CT component of SPECT/CT did not detect any concurrent morphological changes, and the patient exhibited no clinical symptoms. Therefore, an inflammatory etiology for [ ^177^ Lu]Lu-DOTATATE uptake in the gallbladder was highly unlikely. The use of SPECT/CT also allowed us to accurately localize the tracer and provide anatomical morphological features, 9 hence preventing misinterpretation of liver metastasis due to physiological [ ^177^ Lu]Lu-DOTATATE uptake in the gallbladder.

## Conclusion

The identification of tumor lesions via SSTR-PET/CT imaging and the visualization of therapeutic radiopharmaceutical concentration via [ ^177^ Lu]Lu-DOTATATE scintigraphy after PRRT are commonly utilized as theranostic agents in NET. The occurrence of physiological uptake of [ ^177^ Lu]Lu-DOTATATE in the gallbladder is exceedingly uncommon and can be readily misinterpreted as liver metastasis on planar scintigraphy. Utilizing SPECT/CT and understanding the physiological uptake of [ ^177^ Lu]Lu-DOTATATE in the gallbladder enables one to easily prevent the misinterpretation of liver metastasis in such setting.
